# Repeatability of spinal reflexes of lower limb muscles evoked by transcutaneous spinal cord stimulation

**DOI:** 10.1371/journal.pone.0214818

**Published:** 2019-04-04

**Authors:** Akira Saito, Yohei Masugi, Kento Nakagawa, Hiroki Obata, Kimitaka Nakazawa

**Affiliations:** 1 Graduate School of Arts and Sciences, The University of Tokyo, Komaba, Meguro-ku, Tokyo, Japan; 2 Japan Society for the Promotion of Science, Kojimachi, Chiyoda-ku, Tokyo, Japan; 3 Institute of Sports Medicine and Science, Tokyo International University, Matoba, Kawagoe, Saitama, Japan; 4 Department of Humanities and Social Sciences, Institute of Liberal Arts, Kyushu Institute of Technology, Tobata-ku, Kitakyushu, Fukuoka, Japan; University of Ottawa, CANADA

## Abstract

Transcutaneous electrical stimulation is a relatively new technique to evoke spinal reflexes in lower limb muscles. The advantage of this technique is that the spinal reflex responses can be obtained from multiple lower limb muscles simultaneously. However, repeatability of spinal reflexes evoked by transcutaneous spinal cord stimulation between days has not been evaluated. We aimed to examine repeatability of recruitment properties of the spinal reflexes evoked by transcutaneous spinal cord stimulation. Recruitment curves of the spinal reflexes evoked by transcutaneous spinal cord stimulation of 8 lower limb muscles (i.e., foot, lower leg, and thigh muscles) of 20 males were measured on two consecutive days. To confirm that responses were caused by activation of the sensory fiber, a double-pulse stimulation with 50 ms inter-pulse interval was delivered. Peak-to-peak amplitude of the first response was calculated for each muscle when no response was observed in the second response owing to post-activation depression. For comparison with the spinal reflexes evoked by transcutaneous spinal cord stimulation, the recruitment curves of the H-reflex amplitude of the soleus of 9 males were measured. Threshold intensity and maximal slope of the recruitment curves were calculated, and inter-day repeatability of the properties was quantified using intraclass correlation coefficients. For the spinal reflexes evoked by transcutaneous spinal cord stimulation, the intraclass correlation coefficient values of threshold intensity and maximal slope for each muscle ranged from 0.487 to 0.874 and from 0.471 to 0.964, respectively. Regarding the soleus H-reflex, the intraclass correlation coefficients of threshold intensity and maximal slope were 0.936 and 0.751, respectively. The present data showed that repeatability of the recruitment properties of the spinal reflexes evoked by transcutaneous spinal cord stimulation in the lower limb was moderate to high. Measurement of the spinal reflexes evoked by transcutaneous spinal cord stimulation would be useful for longitudinal neurophysiological studies.

## Introduction

The Hoffmann-reflex (H-reflex) is generally used as a measure of a monosynaptic spinal reflex in neurophysiological research [1, 2]. The H-reflex can be produced by transcutaneous electrical stimulation of Ia afferents. Since the measurement of the H-reflex shows good repeatability between days [3, 4], it has been used for longitudinal studies, such as plastic changes in the spinal reflex circuits after strength training [5, 6], motor skill acquisition [7], functional improvement after stroke [8, 9], and spinal cord injury [10]. Although the H-reflex can be evoked from several muscles in the upper and lower limbs (e.g., soleus, SOL; tibialis anterior, TA; flexor carpi radialis, FCR) [3, 11–13], it is difficult to record H-reflex responses for other muscles.

Transcutaneous spinal cord stimulation (tSCS) is an innovative method to evoke spinal reflexes in the lower limb [14–16]. The advantage of this method is that the responses are obtained from multiple lower limb muscles simultaneously [16]. The tSCS mainly activates the posterior roots [16–19], and the evoked responses have characteristics similar to the H-reflex. For instance, the response of the SOL evoked by tSCS was depressed by a prolonged Achilles tendon vibration [15, 20] and strongly depressed by a conditioning stimulus applied 50 ms before the test stimulus [15, 21, 22], similar to the SOL H-reflex [23, 24]. Since the tSCS technique can evoke the spinal reflexes in the lower limb muscles simultaneously, it may be an attractive tool for longitudinal assessment of monosynaptic reflexes in multiple lower limb muscles. However, the repeatability between days of the spinal reflexes evoked by tSCS has not been investigated.

The purpose of this study was to investigate the repeatability of the recruitment properties of spinal reflexes evoked by tSCS. The recruitment curves of spinal reflexes in the lower limb muscles were measured on two consecutive days. In addition, to compare the repeatability of the recruitment properties measured by tSCS with a conventional method, the recruitment curve of the H-reflex from the SOL was also evoked on two consecutive days.

## Methods

### Subjects

Twenty-two males (age, 25.8 ± 3.4 years; height, 173.9 ± 4.6 cm; weight 68.7 ± 9.5 kg) participated in this study. This study consisted of two different measurements: (1) elicitation of spinal reflexes by tSCS in multiple lower limb muscles (*n* = 20); and (2) the H-reflex in the SOL (*n* = 9). Seven subjects underwent both measurements. In each measurement, the subjects visited the laboratory on two consecutive days at approximately the same time each day. The procedure, purpose, risks, and benefits associated with this study were explained to the subjects, and written, informed consent was obtained from all of them. The ethics review committee on experimental research with human subjects of the Graduate School of Arts and Sciences at The University of Tokyo approved the experimental protocols, which were conducted in accordance with the guidelines in the Declaration of Helsinki.

### Surface electromyography recording

Surface electromyographic (EMG) signals were recorded from the flexor digitorum brevis (FDB), extensor digitorum brevis (EDB), TA, SOL, medial head of the gastrocnemius (MG), long head of biceps femoris (BF), vastus medialis (VM), and rectus femoris (RF) in the right lower limb. Prior to attaching the electrodes, the skin was abraded and cleaned with alcohol. Ag-AgCl electrodes (Vitrode F-150S, Nihon Kohden, Japan) with a 20 mm inter-electrode distance were used for EMG acquisition from each muscle. Electrode placement on each muscle was marked after the first session (Day 1), and these electrodes were placed at the same locations at the second session (Day 2). The amplifier was set to a gain of 1000-fold with a bandpass filter between 15 Hz and 3 kHz (AB-611J, Nihon Kohden, Japan). The EMG signals were sampled at 10 kHz and stored on a hard disk over a time period of 100 ms before and 300 ms after electrical stimulation using an AD converter (NI USB-6259, National Instruments, USA) controlled by a custom program (LabVIEW, National Instruments, USA).

### Transcutaneous spinal cord stimulation

Experiments were conducted while subjects were in the supine position. This was because preferential recruitment of the sensory fibers was shown in the supine position compared to the prone and standing positions [17]. The bilateral shoulder, elbow, hip, knee, and ankle joints of the subjects were kept in anatomical positions. Both ankle joints were fixed with ankle-foot orthoses. The subjects were asked to maintain a symmetrical position of the limbs and keep their supine neutral head position as immobile as possible and avoid turning them. An anode (100 mm × 75 mm) was placed over the midline of the abdomen between the xiphoid process of the sternum and the umbilicus and a cathode (50 mm × 50 mm) was placed on the midline of the back between the spinous processes of the upper lumbar vertebrae. Prior to the experiment, cathode placement was determined based on the previous study [14]. The cathodes were placed where larger responses were evoked in the recorded muscles at any stimulation intensity (vertebrae levels: L1/2, *n* = 19; L2/3, *n* = 1), based on visual determination of the response magnitudes. To confirm that the responses were caused by activation of the sensory fibers, a rectangular double-pulse stimulation with 1 ms pulse duration and 50 ms inter-pulse interval was delivered by a constant current electrical stimulator (DS7A, Digitimer, UK) [15, 16]. Stimulation intensity was increased from 2 to 100 mA using 2-mA increments [18], and one stimulus was delivered at each intensity. After Day 1, the placements of the anode and cathode were marked on the skin to facilitate placing them at the same locations on Day 2.

### H-reflex

H-reflex responses of the SOL were evoked by transcutaneous electrical stimulation to the posterior tibial nerve using the electrical stimulator (DS7A, Digitimer, UK) with a single rectangular pulse of 1 ms duration. Subjects maintained the supine position during the experiment in the same way as for tSCS measurement. An anode (50 mm × 50 mm) was placed over the patella and a cathode (10 mm in diameter) was positioned in the popliteal fossa. Stimulation intensity was increased using 0.1-mA increments from approximately 2 mA below the threshold to the maximal H-reflex response (H_max_). Thereafter, the intensity was increased by 1-mA increments until no further increase was observed in the M-wave response (M_max_). One stimulus was delivered at each intensity. The placements of the anode and cathode were marked on the skin after Day 1.

### Data analysis

**Fig 1:**
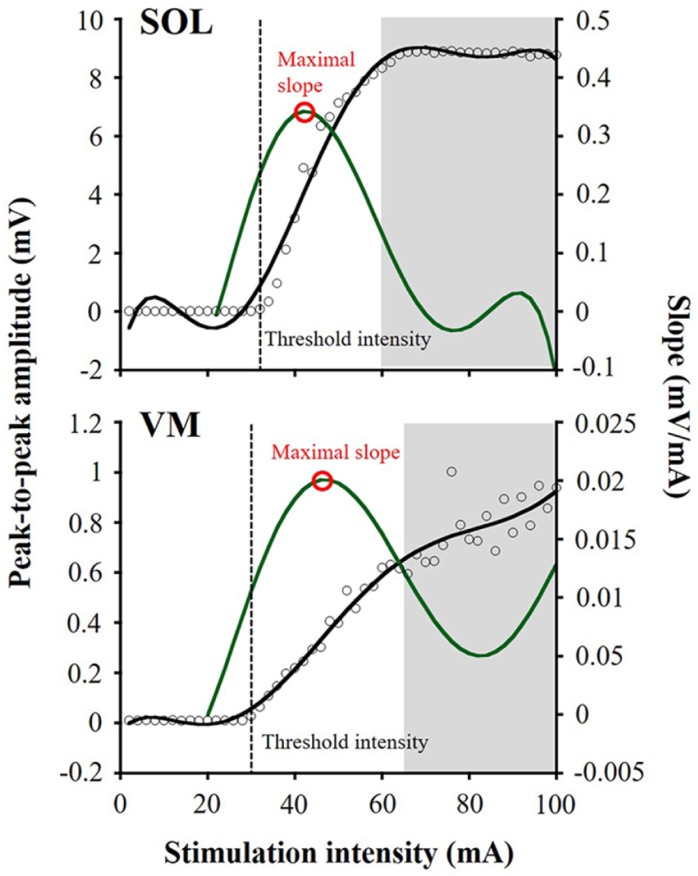
Examples of recruitment curves in the first response recorded from soleus and vastus medialis. Peak-to-peak amplitudes are shown by black open circles (left Y-axis). The amplitudes in the second response that are shown with a grey background were excluded, owing to being out of criteria for the responses. A sixth-order polynomial function (black solid line) is fitted to each recruitment curve and then the slope is calculated as the first derivative (green solid line; right Y-axis). Recruitment properties are taken from the recruitment curve for inter-day comparison: threshold intensity (black dotted line) and maximal slope (red open circle).

Peak-to-peak EMG amplitudes of the evoked responses were calculated for each muscle. Regarding tSCS, this study used two types of exclusion criteria for the responses for further analysis. First, the threshold of the amplitude of the spinal reflexes evoked by tSCS was defined as 100 μV [16]. Based on this definition, the reflexes in the first responses below this threshold were considered as no response when maximal stimulation intensity (i.e., 100 mA) was delivered. Thus, the muscles that showed no response from analysis of the recruitment properties were excluded. Second, if the amplitude of the second response was greater than the threshold (i.e., 100 μV) in a trial, the trial was excluded from calculation of the recruitment properties (Fig 1). This was because the appearance of the second response means that the first response includes the activation of motor fibers [17]. Then, the latency of the first response in each muscle was calculated as the time between the stimulus delivery and the onset of the response. The onset of the spinal reflex was determined by visual assessment of the waveforms [20].

As recruitment properties of spinal reflexes, threshold intensity and maximal slope were calculated (Fig 1) based on the previous study [18]. Threshold intensity was defined as the minimum stimulation intensity that produced the responses with amplitudes greater than the means plus 3 standard deviations (SD) of the baseline values in at least 3 continuous trials. The baseline of each muscle was chosen as the mean value of the first 10 amplitudes, because no response was obtained at the initial 10 trials in all subjects. The maximal slope was determined by fitting a sixth-order polynomial function to the recruitment curve and finding the greatest value of the first derivative.

### Statistics

Inter-day repeatabilities of the recruitment properties and of the latency of the first response were evaluated by intra-class correlation coefficient (ICC), representing relative consistency, and the standard error of measurement (SEM), representing absolute consistency. ICC is sensitive to between-subjects variability, and SEM is an index of the precision or the trial-to-trial noise of the measurement, rather than between-subjects variability [25]. Given that a set time was used between sessions, ICC (3,1) was chosen [12, 26]. According to Versino et al. [27], who examined test-retest repeatability of the H-reflex, ICC was ranked as follows: values < 0.4 can be interpreted as poor repeatability; values between 0.40 and 0.75 can be interpreted as moderate repeatability; and values > 0.75 can be interpreted as excellent repeatability. SEM was calculated with the following equations [28]:

$$SEM=SD\sqrt{1-ICC}$$ (1)

*SD* is the standard deviation across all subjects for each muscle. Statistical analyses were performed using statistical software (IBM SPSS Statistics 24, IBM, Japan).

## Results

**Fig 2:**
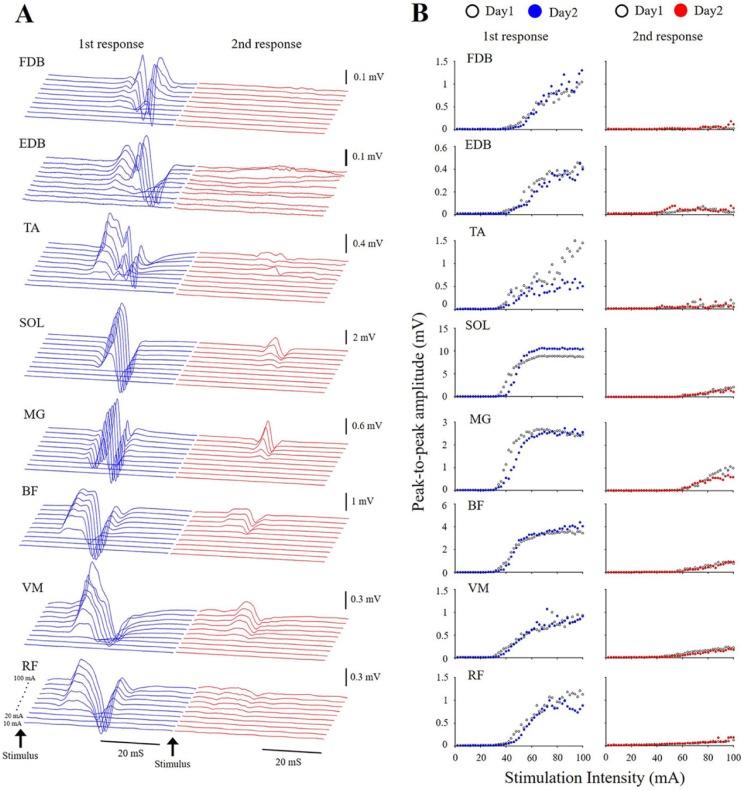
A typical example of recruitment of spinal reflexes evoked by transcutaneous spinal cord stimulation. A: Waveforms of spinal reflexes at different stimulation intensities from 10 to 100 mA. B: Recruitment curves of spinal reflexes between days.

Electrical stimulation over the upper lumbar vertebrae (L1/2 or L2/3 level) induced the spinal reflexes in multiple lower limb muscles. Fig 2 demonstrates a typical example of the evoked potentials at different stimulation intensities with doublet-pulse stimulation (Fig 2A) and recruitment curves of the spinal reflexes in the lower limb (Fig 2B). The latency of the first response of the spinal reflexes was longer in distal than proximal muscles in the lower limb (Table 1). ICC values of the latency of the first response ranged from 0.443 to 0.964, and SEMs ranged from 0.4% to 6.4%. The number of samples differed between the muscles. More specifically, although tSCS induced the spinal reflexes of SOL, MG, and BF muscles successfully in all subjects, spinal reflex responses in some subjects were not observed in the FDB, EDB, TA, VM, and RF in either sessions (Table 1).

**Table 1:** Latency of the first response of the spinal reflexes.

Muscle	Day 1	Day 2	ICC	SEM	%SEM
**FDB (n = 19)**	26.9 ± 1.6	26.6 ± 1.9	0.743	0.462	1.7
**EDB (n = 12)**	24.4 ± 2.5	24.4 ± 2.9	0.906	0.255	1.0
**TA (n = 19)**	17.8 ± 1.9	17.9 ± 1.4	0.676	0.547	3.0
**SOL (n = 20)**	19.1 ± 1.2	18.9 ± 1.2	0.938	0.076	0.4
**MG (n = 20)**	17.5 ± 1.5	17.4 ± 1.4	0.443	0.835	4.7
**BF (n = 20)**	12.3 ± 2.9	12.1 ± 3.3	0.748	0.793	6.4
**VM (n = 19)**	11.0 ± 2.2	11.0 ± 2.2	0.888	0.251	2.2
**RF (n = 14)**	10.4 ± 3.2	10.3 ± 3.3	0.964	0.115	1.1

Values (ms) are the mean and standard deviation. %SEM were divided by the mean value of the latency.

**Fig 3:**
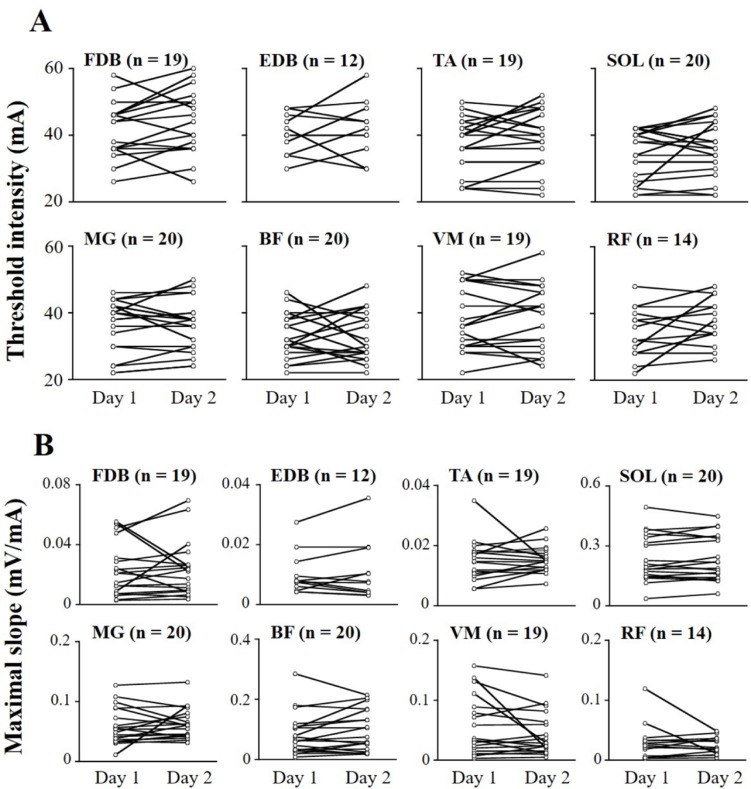
**Threshold intensity (A) and maximal slope (B) of recruitment curves between days.** Each open circle represents a data point in each subject.

Regarding the recruitment properties of individual subjects, threshold intensity and maximal slope between days are shown in Fig 3. ICC values of threshold intensity ranged from 0.487 to 0.874, and SEMs ranged from 8.4% to 14.7% (Table 2). ICCs of maximal slope ranged from 0.471 to 0.964, and SEMs ranged from 9.1% to 63.0%.

**Table 2:** Inter-day repeatability of recruitment properties using transcutaneous spinal cord stimulation.

	Threshold intensity (mA)			Maximal slope (mV/mA)		
Muscle	ICC	SEM	%SEM	ICC	SEM	%SEM
**FDB (n = 19)**	0.701	5.075	11.6	0.673	0.010	44.1
**EDB (n = 12)**	0.487	4.889	11.9	0.904	0.002	24.5
**TA (n = 19)**	0.807	3.742	9.8	0.474	0.003	25.8
**SOL (n = 20)**	0.702	4.166	11.9	0.964	0.021	9.1
**MG (n = 20)**	0.843	3.070	8.4	0.627	0.016	27.9
**BF (n = 20)**	0.522	4.883	14.7	0.845	0.026	30.4
**VM (n = 19)**	0.874	3.413	8.7	0.667	0.024	50.6
**RF (n = 14)**	0.568	4.772	13.3	0.471	0.017	63.0

%SEM were divided by the mean value of recruitment properties.

Regarding the recruitment properties of the SOL H-reflex, the ICC of the threshold intensity was 0.936, and the SEM was 11.0% (Table 3). The ICC of the maximal slope was 0.751, and the SEM was 18.4%.

**Table 3:** Inter-session repeatability of recruitment properties of the H-reflex.

	Threshold intensity (mA)			Maximal slope (mV/mA)		
Muscle	ICC	SEM	%SEM	ICC	SEM	%SEM
**SOL (n = 9)**	0.936	0.903	11.0	0.751	0.327	18.4

%SEM were divided by the mean value of recruitment properties.

## Discussion

This study aimed to investigate the repeatability of the recruitment properties of spinal reflexes evoked by tSCS. The main finding of this study was that inter-day repeatability of the recruitment properties was moderate to high.

It is considered that the spinal reflexes evoked by tSCS involve the activation of sensory (posterior root), motor (anterior root), or mixed sensory-motor fibers [17]. The nature of these responses has been identified using paired-pulse stimulation, based on suppression of the second response owing to post-activation depression [14–16, 24]. The depression of the second response could be primarily caused by homosynaptic depression and secondarily by heteronymous inhibitory pathways, which induce Ia afferent terminals leading to a transient reduction in neurotransmitter release [14]. In this study, a trial that showed the second response was excluded from the analysis (Fig 1). Thus, recruitment curves obtained in this study reflect the properties of the spinal reflexes in each muscle. The results showed that the ICC values of the threshold intensity and maximal slope of the recruitment curves measured by tSCS between days ranged from moderate to high (Table 2). The measurements of threshold intensity and maximal slope of the recruitment curve provide the minimum inputs to sensory afferents onto the moto-neuron pools [18] and the characteristics of the recruitment of afferents projecting onto the moto-neuron pool [29], respectively. Therefore, the present results suggest that tSCS could provide good to excellent repeatability of the neural circuits in sensory-motor pathways.

In many cases, the ICC values of the recruitment properties measured by tSCS were lower than those of the SOL H-reflex (ICC > 0.75, Table 3). Differences in the stimulation techniques between tSCS and the H-reflex would induce the discrepancy of repeatability results. More specifically, tSCS activates the posterior root fibers at multiple spinal levels [17, 18], whereas the H-reflex activates the sensory fibers of the posterior tibial nerve [1]. In addition to the activation of spinal roots, the spinal interneurons may be gradually recruited with increasing stimulation intensity [29, 30]. Hence, such concomitant activation with the spinal roots may affect the variability of the spinal reflexes. Moreover, a higher SEM of the recruitment properties was obtained by tSCS than the H-reflex in most muscles (Tables 2 and 3). The SEM is an index of the absolute trial-to-trial error across the sessions [25]. Thus, measurement error of tSCS between days is one of the factors inducing the discrepancy in repeatability of the recruitment properties between tSCS and the H-reflex. However, this study could not resolve such possible mechanisms.

In this study, stimulating electrode (i.e., cathode) placement was mostly located between the L1 and L2 spinous processes because this cathode location provided the largest response from multiple lower limb muscles. Nevertheless, no spinal reflex response (e.g., RF muscle) was obtained in some subjects when maximal stimulation intensity was delivered (Table 1 and Fig 3). Anatomically, the RF and SOL are innervated by L2-L4 and S1-S2 nerve roots, respectively. Thus, the relationship between stimulating electrode location and the anatomical structure of muscle innervation might affect the amplitudes of the spinal reflex response of each muscle.

Stimulating electrode placement of tSCS affects the magnitude and recruitment properties of the spinal reflexes in the lower limb [14, 18]. Inter-day variability of stimulating electrode placements for tSCS may affect the relatively lower repeatability of the tSCS results. However, the cathodes of tSCS and EMG electrodes in this study were kept at the same location across the sessions by skin marking. Instead, differences of dorsal vertebral alignment of the subjects may affect the repeatability across the sessions in multiple lower limb muscles.

Methodological limitations to repeatability evaluation of the recruitment properties measured by tSCS between days should be considered in this study. The previous studies recommended that more than 3 trials are needed to improve the accuracy of the H-reflex measurement [4, 26]. Unfortunately, only one stimulus current was delivered at each intensity to evoke the spinal reflexes using tSCS, due to subjects feeling uncomfortable with stimulation at a higher intensity. Therefore, inter-day repeatability of the recruitment properties in the lower limb muscles may be improved if these limitations were resolved.

In conclusion, this study demonstrated that moderate to high repeatability of the recruitment properties of the spinal reflexes evoked by tSCS was obtained in multiple lower limb muscles between two consecutive days. Measurement of the spinal reflexes evoked by tSCS appears to be an attractive tool for longitudinal neurophysiological studies to evaluate the neural circuitry responses in sensory-motor pathways.
